# Forecasting Hospital Room and Ward Occupancy Using Static and Dynamic Information Concurrently: Retrospective Single-Center Cohort Study

**DOI:** 10.2196/53400

**Published:** 2024-03-21

**Authors:** Hyeram Seo, Imjin Ahn, Hansle Gwon, Heejun Kang, Yunha Kim, Heejung Choi, Minkyoung Kim, Jiye Han, Gaeun Kee, Seohyun Park, Soyoung Ko, HyoJe Jung, Byeolhee Kim, Jungsik Oh, Tae Joon Jun, Young-Hak Kim

**Affiliations:** 1 Department of Medical Science Asan Medical Institute of Convergence Science and Technology Asan Medical Center & University of Ulsan College of Medicine Seoul Republic of Korea; 2 Department of Information Medicine Asan Medical Center Seoul Republic of Korea; 3 Division of Cardiology Asan Medical Center Seoul Republic of Korea; 4 Department of Digital Innovation Asan Medical Center Seoul Republic of Korea; 5 Big Data Research Center Asan Institute for Life Sciences Asan Medical Center Seoul Republic of Korea; 6 Division of Cardiology Department of Information Medicine Asan Medical Center & University of Ulsan College of Medicine Seoul Republic of Korea

**Keywords:** hospital bed occupancy, electronic medical records, time series forecasting, short-term memory, combining static and dynamic variables

## Abstract

**Background:**

Predicting the bed occupancy rate (BOR) is essential for efficient hospital resource management, long-term budget planning, and patient care planning. Although macro-level BOR prediction for the entire hospital is crucial, predicting occupancy at a detailed level, such as specific wards and rooms, is more practical and useful for hospital scheduling.

**Objective:**

The aim of this study was to develop a web-based support tool that allows hospital administrators to grasp the BOR for each ward and room according to different time periods.

**Methods:**

We trained time-series models based on long short-term memory (LSTM) using individual bed data aggregated hourly each day to predict the BOR for each ward and room in the hospital. Ward training involved 2 models with 7- and 30-day time windows, and room training involved models with 3- and 7-day time windows for shorter-term planning. To further improve prediction performance, we added 2 models trained by concatenating dynamic data with static data representing room-specific details.

**Results:**

We confirmed the results of a total of 12 models using bidirectional long short-term memory (Bi-LSTM) and LSTM, and the model based on Bi-LSTM showed better performance. The ward-level prediction model had a mean absolute error (MAE) of 0.067, mean square error (MSE) of 0.009, root mean square error (RMSE) of 0.094, and R^2^ score of 0.544. Among the room-level prediction models, the model that combined static data exhibited superior performance, with a MAE of 0.129, MSE of 0.050, RMSE of 0.227, and R^2^ score of 0.600. Model results can be displayed on an electronic dashboard for easy access via the web.

**Conclusions:**

We have proposed predictive BOR models for individual wards and rooms that demonstrate high performance. The results can be visualized through a web-based dashboard, aiding hospital administrators in bed operation planning. This contributes to resource optimization and the reduction of hospital resource use.

## Introduction

### Background

The global health care market continues to grow, but the burden of health care costs on governments and individuals is reaching its limits. Consequently, there is increasing interest in the efficient use of limited resources in health care systems, and hospitals must develop approaches to maximize medical effectiveness within budgetary constraints [1,2]. One approach to this is optimizing the use of medical resources. Medical resources can be broadly categorized into 3 categories: human resources, physical capital, and consumables. The appropriate and optimized use of these resources is critical for improving health care quality and providing care to a larger number of patients [3,4].

Among the 3 medical resources, hospital beds are considered one of the physical capitals provided by hospitals to patients. These beds are allocated for various purposes, such as rest, hospitalization, postsurgical recovery, etc. They constitute one of the factors that can directly influence the patient’s internal satisfaction within the hospital. However, owing to limited space, hospitals often have a restricted number of beds. Moreover, the number and functionality of beds are often fixed owing to budgetary or environmental constraints, making it difficult to make changes. Nonetheless, if hospital administrators can evaluate bed occupancy rates (BORs) according to different time periods, they can predict the need for health care professionals and resources. On the basis of this information, hospitals can plan resources efficiently, reduce operational costs, and achieve economic objectives [5]. In addition, excessive BORs can exert a negative effect on the health of staff members and increase the possibility of exposure to infection risks. Hence, emphasizing only maintaining a high BOR may not necessarily lead to favorable outcomes for the hospital [6,7]. Considering these reasons, BOR prediction plays a vital role in hospitals and is recognized as a broadly understood necessity for resource optimization in the competitive medical field.

In the medical field, optimizing resources is crucial in the face of limited bed capacity and intense competition. Therefore, bed planning is a vital consideration aimed at minimizing hospital costs [8]. To achieve this, hospitals need to plan staffing and vacations weeks or months in advance [9]. The use of machine learning (ML) technology for BOR prediction is necessary to address fluctuations in patient numbers due to seasonal variations or infectious diseases, ensuring continuous hospital operations. In the Netherlands, hospitals have already implemented ML-based BOR prediction [10], and Johns Hopkins Hospital uses various metrics to effectively manage bed capacity for optimization. Predicting BORs based on quantitative data contributes to validating the clinical quality and cost-effectiveness of treatments. This, in turn, enhances overall accountability throughout the wards and contributes to improving hospital efficiency [11].

### Prior Work

Hospital BOR prediction has been investigated using various approaches recently. From studies predicting bed demand using mathematical statistics or regression equation models based on given data [12-15], the focus has shifted toward modeling approaches using time-series analysis. This approach observes recorded data over time to predict future values.

A previous study has taken an innovative approach using time-series analysis alongside the commonly used regression analysis for bed demand prediction, and the study demonstrated that using time-series prediction for bed occupancy yielded higher performance results than using a simple trend fitting approach [16]. Another study used the autoregressive integrated moving average (ARIMA) model for univariate data and a time-series model for multivariate data to predict BORs [17]. With the advancement of deep learning (DL) models that possess strong long-term memory capabilities, such as recurrent neural network (RNN) and long short-term memory (LSTM), there has been an increase in studies applying these models to time-series data for prediction purposes. For instance, in the study by Kutafina et al [9], hospital BORs were predicted based on dates and public holiday data from government agencies and schools, without involving the personal information of patients. The study used a nonlinear autoregressive exogenous model to predict a short-term period of 60 days, with an aim to contribute to the planning of hospital staff. The model demonstrated good performance, with an average mean absolute percentage error of 6.24%. In emergency situations, such as the recent global COVID-19 pandemic, the sudden influx of infected patients can disrupt the hospitalization plans for patients with pre-existing conditions [18]. Studies have been conducted using DL architectures to design models for predicting the BOR of patients with COVID-19 on a country-by-country basis. Some studies incorporated additional inputs, such as vaccination rate and median age, to train the models [19]. Studies have also been conducted to focus on the short-term prediction of BORs during the COVID-19 period [20,21]. Prior studies are summarized in Table 1.

Although previous research has contributed to BOR prediction and operational planning at the hospital level, more detailed and systematic predictions are necessary for practical application in real-world operations. To address this issue, studies have developed their own computer simulation hospital systems to not only predict bed occupancy but also execute scheduling for admissions and surgeries to enhance resource utilization [22-24]. Nevertheless, existing studies have the limitation of focusing solely on the overall BOR of the hospital. As an advancement to these studies, we aim to propose a strategy for predicting the BOR at the level of each ward and room using various variables in a time-series manner. Interestingly, to our knowledge, this is the first study to apply DL to predict ward- and room-specific occupancy rates using time-series analysis.

**Table 1:** Summary of prior studies.

Study	Year	Data set	Method	Prediction target
Mackay and Lee [12]	2007	Deidentified data, the date and time of patient admission and discharge between 1998 and 2000	Comparison of 2 compartment models through cross-validation	Entire hospital bed occupancy (annual average)
Littig and Isken [13]	2007	Historical and real-time data warehouse and hospital information systems (emergency department, financial, surgical scheduling, and inpatient tracking systems)	Computerized model of MLR^a^ and LR^b^	Entire hospital short-term occupancy (24 h or 72 h) based on LOS^c^
Kumar and Mo [14]	2010	Bed management between June 1, 2006, and June 1, 2007; Information: (1) In each class based on length of stay and admission data; (2) Historical previous year’s same week admission data; (3) Relationship between identified variables to aid bed managers	The 3 methods are: (1) Poisson bed occupancy model; (2) Simulation model; and (3) Regression model	The 3 prediction targets are: (1) Estimation of bed occupancy and optimal bed requirements in each class; (2) Bed occupancy levels for every class for the following week; and (3) Weekly average number of occupied beds
Seematter-Bagnoud et al [15]	2015	Inpatient stay data in 2010 (acute somatic care inpatients and outpatients)	Three models of hypothesis-based statistical forecasting of future trends	The 3 targets are: (1) Number of hospital stays; (2) Hospital inpatient days; and (3) Beds for medical stay
Farmer and Emami [16]	1990	Inpatient stay data for general surgery in the age group of 15-44 years between 1969 and 1982	The 2 methods are: (1) Forecasting from a structural model and (2) The time-series or Box-Jenkins method	Entire hospital short-term daily bed requirements
Kim et al [17]	2014	Data warehouse between January 2009 and June 2012	The 2 methods are: (1) The ARIMA^d^ model for univariate data and (2) The time-series model for multivariate data	Entire hospital bed occupancy (1 day and 1 week)
Kutafina et al [9]	2019	Inpatient stay data between October 14, 2002, and December 31, 2015 (patient identifier, time of admission, discharge, and name of the clinic the patient was admitted to; no personal information on the patients or staff was provided)	NARX^e^ model, a type of RNN^f^	Entire hospital mid-term bed occupancy (60 days, bed pool in units of 30 beds)
Bouhamed et al [19]	2022	COVID-19 hospital occupancy data in 15 countries between December 2021 and early January 2022	The 3 models are: LSTM^g^, GRU^h^, and SRNN^i^. Incorporate vaccination percentage and median age of the population to improve performance	Entire hospital bed occupancy
Bekker et al [20]	2021	Historical data publicly available until mid-October 2020	The 2 methods are: (1) Using linear programming to predict admissions and (2) Fitting the remaining LOS and using results from the queuing theory to predict occupancy	The 2 targets are: (1) Patient admission and (2) Entire hospital short-term bed occupancy
Farcomeni et al [21]	2021	Patients admitted to the intensive care unit between January and June 2020	The 2 methods are: (1) Generalized linear mixed regression model and (2) Area-specific nonstationary integer autoregressive methodology	Entire hospital short-term intensive care bed occupancy

^a^MLR: multinomial logistic regression.

^b^LR: linear regression.

^c^LOS: length of stay.

^d^ARIMA: autoregressive integrated moving average.

^e^NARX: nonlinear autoregressive exogenous.

^f^RNN: recurrent neural network.

^g^LSTM: long short-term memory.

^h^GRU: grid recurrent unit.

^i^SRNN: simple recurrent neural network.

### Goal of This Study

The aim of this study was to predict the BORs of hospital wards and rooms using time-series data from individual beds. Although overall bed occupancy prediction is useful for macro-level resource management in hospitals, resource allocation based on the prediction of occupancy rates for each ward and room is required for specific hospital scheduling and practicality. Through this approach, we aim to contribute to the efficient operational cost optimization of the hospital and ensure the availability of resources required for patient care.

We have developed time-series prediction models based on deep neural network (DNN), among which 1 model combines data representing room-specific features (static data) with dynamic data to enhance the prediction performance for room bed occupancy rates (RBORs). Based on bidirectional long short-term memory (Bi-LSTM), the RBOR prediction model demonstrates a lower mean absolute error (MAE) of 0.049, a mean square error (MSE) of 0.042, a root mean square error (RMSE) of 0.007, and a higher R^2^ score of 0.291, indicating the highest performance among all RBOR models.

We developed 6 types of BOR prediction models, of which 2 types were used for predicting ward bed occupancy rates (WBORs), and the other 4 types focused on predicting RBORs. These models use LSTM and Bi-LSTM architectures with strong long-term memory capabilities as their basic structure. We created 6 models for each architecture, resulting in a total of 12 models. The WBOR models were used for predicting weekly and monthly occupancy rates, serving long-term hospital administrative planning purposes. Conversely, the RBOR models were designed for immediate and rapid occupancy planning and were trained with 3- and 7-day intervals. Each RBOR model was enhanced by combining static data, which represent room-specific features, to generate more sophisticated prediction models.

**Figure 1:**
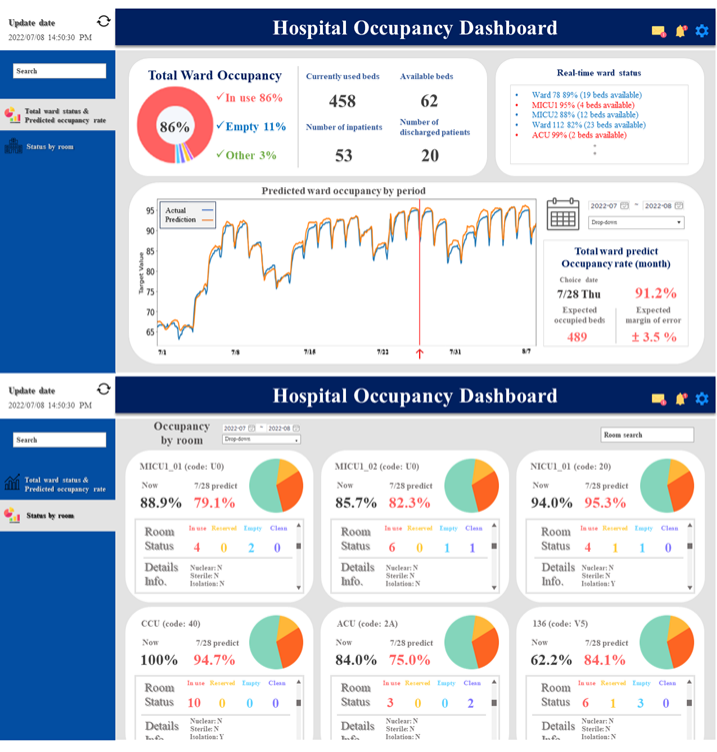
Virtual dashboard of the status and forecast of the ward bed occupancy rate (WBOR) and room bed occupancy rate (RBOR). The first screen presents the overall bed occupancy rate of the hospital, along with the number of beds in use and available. Moreover, a predictive graph displays the anticipated WBOR for selected dates. The second screen presents the WBOR for individual beds, indicating their statuses, such as “in use,” “reserved,” “empty,” and “cleaning.” Detailed information about each room is also displayed.

Figure 1 shows the potential application of our model as a form of web software in a hospital setting. Through an online dashboard, it can provide timely information regarding bed availability, enabling intelligent management of patient movements related to admission and discharge. It facilitates shared responsibilities within the hospital and simplifies future resource planning [25].

In the Introduction section, we explored the importance of this research and investigated relevant previous studies, providing a general overview of the direction of our research. In the Methods section, we provide descriptions of the data set used and the structure of the DNN algorithm used, and explain the model architecture and performance. In the Results section, we present the performance and outcomes of this study. Finally, in the Discussion section, we discuss the contributions, limitations, and potential avenues for improvement of the research.

## Methods

### Overview

We intended to predict the BORs of individual hospital wards and rooms based on the information accumulated in individual bed–level data on an hourly basis, aggregated on a daily basis. For this purpose, we developed 12 time-series models. As the base models, we applied LSTM and Bi-LSTM, which are suitable for sequence data. These models address the limitation of long-term memory loss in traditional RNNs and were chosen because of their suitability for training bed data represented as sequence data.

Based on the model architecture, there were 2 WBOR prediction model types, which were trained at 7- and 30-day intervals to predict the occupancy rate for the next day. Moreover, there were 2 RBOR prediction model types, similar to the ward models, which were trained at 3- and 7-day intervals. Furthermore, as another approach, each RBOR prediction model was augmented with static data, and 2 DL algorithms were proposed for the final comparison of their performances in predicting RBORs.

### Ethical Considerations

The study was approved by the Asan Medical Center (AMC) Institutional Review Board (IRB 2021-0321) and was conducted in accordance with the 2008 Declaration of Helsinki.

### Materials

#### Study Setting

This was a retrospective single-center cohort study. Data were collected from AMC, with information on the occupancy status of each bed recorded at hourly intervals between May 27, 2020, and November 21, 2022. The data set comprised a total of 54,632,684 records. This study used ethically preapproved data. Deidentified data used in the study were extracted from ABLE, the AMC clinical research data warehouse.

A total of 57 wards, encompassing specialized wards; 1411 rooms, including private and shared rooms; and 4990 beds were included in this study. Wards and rooms with specific characteristics, such as intensive care unit, newborn room, and nuclear medicine treatment room, were excluded from the analysis as their occupancy prediction using simple and general variables did not align with the direction of this study.

#### Supporting Data

Supporting data for public holidays were added in our data set. We considered that holidays have both a recurring pattern with specific dates each year and a distinctive characteristic of being nonworking days, which could affect occupancy rates. Based on Korean public holidays, which include Chuseok, Hangeul Proclamation Day, Children’s Day, National Liberation Day, Memorial Day, Buddha’s Birthday, Independence Movement Day, and Constitution Day, there were 27 days that corresponded to public holidays during the period covered by the data set. We denoted these dates with a value of “1” if they were public holidays and “0” if they were not, based on the reference date.

#### Preprocessing and Description of Variables

Among the variables representing individual beds, the reference date, ward and room information, patient occupancy status, bed cleanliness status, and detailed room information were available. Based on the recorded date of bed status, we derived additional variables, such as the reference year, reference month, reference week (week of the year), reference day, and reference day of the week.

Room data were derived from the input information representing the cleanliness status of beds. This variable had 2 possible states, namely, “admittable” and “discharge.” If neither of these states was indicated, it implied that a patient was currently hospitalized in the bed. As the status of hospitalized patients was indicated by missing values, we replaced them with the number “1” to indicate the presence of a patient in the bed and “0” otherwise. The sum of all “1” values represented the current number of hospitalized patients. The count of beds in each room indicated the capacity of each room. The target variable BOR was calculated by dividing the number of patients in the room by the room capacity, resulting in a room-specific patient occupancy rate variable. The ward data were subjected to a similar process as that of the room data, with the difference being that we generated ward-specific variables, such as ward capacity and WBOR, using the same approach. The static room data consisted of 14 variables, including the title of the room and the detailed information specific to each room.

For the variables in the ward and room data, we disregarded the units of the features and converted them into numerical values for easy comparison, after which we performed normalization. Regarding the variables representing detailed room information, we converted them to numerical values where “yes” was represented as “1” and “no” was represented as “0.”

The final set of variables used in this study was categorized into date, ward, room, and detailed room information. Table 2 provides the detailed descriptions of the variables used in our training, including all the administrative data related to beds that are readily available in the hospital.

The explanation of the classification for generating the data sets for training each model is provided in Table 3. The static features of the detailed room information were combined with the room data set, which has sequence characteristics, to generate a separate data set termed Room+Static.

**Table 2:** Description of variables by category.

Variable			Type		Description
**Date**					
	Year	3 categories		Reference year for bed status	
	Month	12 categories		Reference month for bed status	
	Week	53 categories		Reference week for bed status	
	Day	31 categories		Reference day for bed status	
	Weekday	7 categories		Reference day of the week for bed status	
	Holiday	2 categories		Holiday status	
**Ward**					
	Ward abbreviation	57 categories		Abbreviations for entire ward names	
	Ward capacity	Numeric		Number of available ward beds	
	Ward bed capacity	Numeric		Number of patients currently admitted to the ward	
	Ward occupancy rate	Numeric		Ward bed capacity divided by ward capacity	
**Room**					
	Room abbreviation	1411 categories		Abbreviations for entire room names	
	Room capacity	Numeric		Number of available room beds	
	Room bed capacity	Numeric		Number of patients currently admitted to the room	
	Room occupancy rate	Numeric		Room bed capacity divided by room capacity	
**Room static feature**					
	Room code	34 categories		Room grade code	
	Nuclear	2 categories (N^a^/Y^b^)		Nuclear medicine room availability	
	Sterile	2 categories (N/Y)		Sterile room availability	
	Isolation	2 categories (N/Y)		Isolation room availability	
	EEG^c^ testing	2 categories (N/Y)		EEG testing room availability	
	Observation	2 categories (N/Y)		Observation room availability	
	Kidney	2 categories (N/Y)		Kidney transplant room availability	
	Liver	2 categories (N/Y)		Liver transplant room availability	
	Sub-ICU^d^	2 categories (N/Y)		Sub-ICU room availability	
	Special	2 categories (N/Y)		Special room availability	
	Small single	2 categories (N/Y)		Small single room availability	
	Short-term	2 categories (N/Y)		Short-term room availability	
	Psy-double	2 categories (N/Y)		Psychiatry department double room availability	
	Psy-open	2 categories (N/Y)		Psychiatry department open room availability	

^a^N: No.

^b^Y: Yes.

^c^EEG: electroencephalogram.

^d^ICU: intensive care unit.

**Table 3:** Data set classification and included variables.

Data set	Variables
Ward data set	Ward abbreviation, year, month, week, day, weekday, holiday, ward capacity, ward bed capacity, and ward occupancy rate
Room data set	Room abbreviation, year, month, week, day, weekday, holiday, room capacity, room bed capacity, and room occupancy rate
Static data set	14 static variables related to detailed room information
Room+Static data set	Room abbreviation, year, month, week, day, weekday, holiday, room capacity, room bed capacity, 14 static variables related to detailed room information, and room occupancy rate

#### Separation

Each data set was split into training, validation, and test sets for training and evaluation of the model. The training set consisted of 32,153 rows (67.8%), with data from May 27, 2020, to December 2021. The validation set, used for parameter tuning, included 7085 rows (15.0%), with data from January to June 2022. Finally, the test set comprised 8208 rows (17.2%), with data from July 2022 to November 21, 2022.

### DL Algorithms

We used various DL algorithms for in-depth learning. In the following subsections, we will provide explanations for each model algorithm used in our research.

#### LSTM Network

RNN [26] is a simple algorithm that passes information from previous steps to the current step, allowing it to iterate and process sequential data. However, it encounters difficulties in handling long-term dependencies, such as those found in time-series data, owing to the vanishing gradient problem. To address this issue, LSTM [27] was developed. LSTM excels in handling sequence data and is commonly used in natural language processing, machine translation, and time-series data analysis. LSTM consists of an input gate, output gate, and forget gate. The “cell state,” is carefully controlled by each gate to determine whether the memory should be retained or forgotten for the next time step.

#### Bi-LSTM Network

Although RNN and LSTM possess the ability to remember previous data, they have a limitation in that their results are primarily based on immediate past patterns because the input is processed in a sequential order. This limitation can be overcome through a network architecture known as Bi-LSTM [28]. Bi-LSTM allows end-to-end learning, minimizing the loss on the output and simultaneously training all parameters. It also has the advantage of performing well even with long data sequences. Because of its suitability for models that require knowledge of dependencies from both the past and future, such as LSTM-based time-series prediction, we additionally selected Bi-LSTM as the base model.

#### Attention Mechanism

Attention mechanism [29,30] refers to the process of incorporating the encoder’s outputs into the decoder at each time step of predicting the output sequence. Rather than considering the entire input sequence, it focuses more on the relevant components that are related to the predicted output, allowing the model to focus on important areas. This mechanism helps minimize information loss in data sets with long sequences, enabling better learning and improving the model’s performance. It has been widely used in areas such as text translation and speech recognition. Nevertheless, as it is still based on RNN models, it has the drawbacks of slower speed and not being completely free from information loss issues.

#### Combining Static and Dynamic Features

Data can exhibit different characteristics even at the same time. For instance, in data collected at 1-hour intervals for each hospital bed, we can distinguish between “dynamic data,” which include features that change over time, such as the bed condition, date, and patient occupancy, and “static data,” which consist of information that remains constant, such as the ward and room number.

DL allows us to use all the available information for prediction. Therefore, for predicting the RBOR, we investigated an approach that combines dynamic and static data using an LSTM-based method [31]. This approach demonstrated better performance than LSTM alone [32]. Our approach involves adding a layer that incorporates static data as an input to the existing room occupancy prediction model.

### Model Architecture

#### Base Model

Our objective was to predict the intermediate-term occupancy rates of wards and rooms within the hospital to contribute to hospital operation planning. Bi-LSTM was chosen as the base model owing to its improved predictive performance compared with the traditional LSTM model. However, to quantitatively compare these models, we conducted a comparison of the results for each model (6 for each, with a total of 12 models).

A typical LSTM model processes data sequentially, considering only the information from the past up to the current time step. However, Bi-LSTM, by simultaneously processing data in both forward and backward directions, has a unique feature that allows it to leverage both current and future information for predictions. This bidirectionality helps the model effectively learn temporal dependencies and intricate patterns. However, despite these advantages, Bi-LSTM comes with the trade-off of doubling the number of model parameters, resulting in increased computational costs for training and prediction. While a more complex model can better adapt to the training data, there is an increased risk of overfitting, especially with small data sets. Nevertheless, the reason for choosing Bi-LSTM for tasks like predicting BORs in hospitals, involving time-series data, lies in its ability to harness the power of bidirectional information. Bi-LSTM processes input data from both past and future directions simultaneously, enabling it to effectively incorporate future information into current predictions. This proves beneficial for handling complex patterns in long time-series data [28].

Moreover, we have enhanced the performance of our models by adding an attention layer to Bi-LSTM. The attention layer assigns higher weights to features that exert a significant impact on the prediction, allowing the model to focus on relevant information and gather necessary input features. This helps improve the accuracy of the prediction. Furthermore, the attention layer reduces the amount of information processed, resulting in improved computational efficiency. Ultimately, this contributes toward enhancing the overall performance of the model.

**Figure 2:**
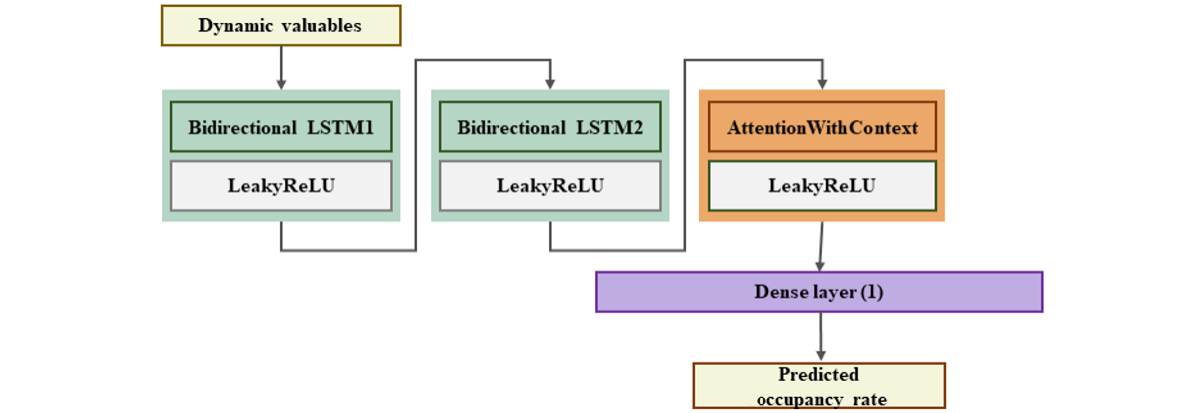
Base bidirectional long short-term memory (Bi-LSTM) model architecture. LeakyReLU: leaky rectified linear unit; LSTM: long short-term memory.

The window length of the input sequence was divided into 3 different intervals, namely, 3, 7, and 30 days. The WBOR model was trained on sequences with a window length of 7 and 30 days, whereas the RBOR model was trained on sequences with a window length of 3 and 7 days. The first layer of our model consisted of Bi-LSTM, which was followed by the leaky rectified linear unit (LeakyReLU) activation function. LeakyReLU is a linear function that has a small gradient for negative input values, similar to ReLU. It helps the model converge faster. After applying this process once again, the AttentionWithContext layer was applied, which focuses on important components of input sequence data and transforms outputs obtained from the previous layer. After applying the activation function again, a dense layer with 1 neuron was added for generating the final output. The sigmoid function was used to limit the output values between 0 and 1. Finally, our model was compiled using the MSE loss function, Adam optimizer, and MAE metric. The parameters for each layer were selected based on accumulated experience through research. Figure 2 visually represents the above-described structure.

#### Combining Dynamic and Static Data Using the DL Model

The accumulated bed data, which were collected on a time basis, were divided into dynamic and static data of the rooms, which were then inputted separately. To improve the performance of the BOR prediction model, we designed different DL architectures for the characteristics of these 2 types of data.

We first used a base model based on LSTM and Bi-LSTM to learn the time-series data and then focused the model’s attention using the dense layer to process fixed-size inputs. To prevent overfitting, we applied the dropout function to randomly deactivate neurons in 2 dense layers. The hidden states of the 2 networks were combined, and the resulting output was passed to a single layer, combining the time dynamic and static data.

**Figure 3:**
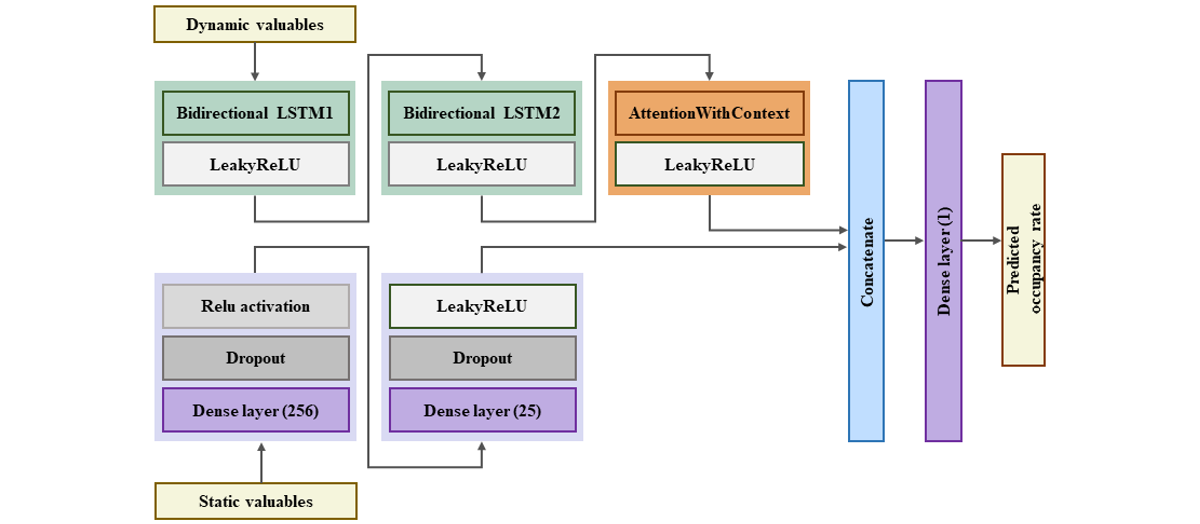
Bidirectional long short-term memory (Bi-LSTM) model architecture combining static and dynamic variables. LeakyReLU: leaky rectified linear unit; LSTM: long short-term memory.

Finally, the hidden states of the 2 networks were combined, and the combined result was passed to a single layer to effectively integrate the dynamic and static data. This allowed us to use the information from both the dynamic and static data for BOR prediction. This architecture is illustrated in Figure 3.

### Hyperparameter Tuning

One of the fundamental methods to enhance the performance of artificial intelligence (AI) learning models is the use of hyperparameter tuning. Hyperparameters are parameters passed to the model to modify or adjust the learning process. While hyperparameter tuning may rely on the experience of researchers, there are also functionalities that automatically search for hyperparameters, taking into account the diversity of model structures.

Various methods for search optimization have been proposed [33,34], but we implemented our models using the Keras library. By leveraging Keras Tuner, we automatically searched for the optimal combinations of units and learning rates for each model, contributing to the improvement of their performance.

### Time Series Cross-Validation

Time-series data exhibit temporal dependencies between data points, making it crucial to consider these characteristics when validating a model. Commonly used K-fold cross-validation is effective for evaluating models on general data sets [35], providing effectiveness in preventing overfitting and enhancing generalizability by dividing the data into multiple subsets [36,37]. However, for time-series data, shuffling the data randomly is not appropriate owing to the inherent sequential dependency of the observations.

Time series cross-validation is a method that preserves this temporal dependence while dividing the data [38]. It involves splitting the entire hospital bed data set into 5 periods, conducting training and validation for each period, and repeating this process as the periods shift. This approach is particularly effective when observations in the dynamic data set, such as hospital bed data recorded at 1-hour intervals, play a crucial role in predicting future values based on past observations.

Shuffling data randomly using K-fold may disrupt the temporal continuity, leading to inadequate reflection of past and future observations. Therefore, time series cross-validation sequentially partitions the data, ensuring the temporal flow is maintained, and proves to be more effective in evaluating the model’s performance. This method enables the model to make more accurate predictions of future occupancy based on past trends.

#### Evaluation

We selected various metrics to evaluate the performance of time-series data predictions. Among them, MAE represents the absolute difference between the model’s predicted values and the actual BOR. We also considered MSE, which is sensitive to outliers. Moreover, to address the limitations of MSE and provide a penalty for large errors, we opted for RMSE. We also used the R^2^ score to measure the correlation between the predicted and actual values.

MAE is a commonly used metric to evaluate the performance of time-series prediction models. MAE is intuitive and easy to calculate, making it widely used in practice. Because MAE uses absolute values, it is less sensitive to outliers in the occupancy rate values for specific dates. MAE is calculated using the following formula:



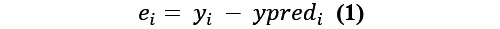





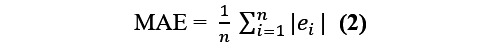



MSE is a metric that evaluates the magnitude of errors by squaring the differences between the predicted and actual values and then taking the average. It is calculated using the following formula:



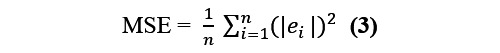



RMSE is used to address the limitations of MSE where the error scales as a square, providing a more intuitive understanding of the error magnitude between the predicted and actual values. It penalizes large errors, making it less sensitive to outliers. RMSE is calculated using the following formula:



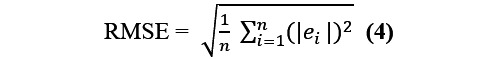



The R^2^ score is used to measure the explanatory potential of the prediction model, and it is calculated using the following formula:



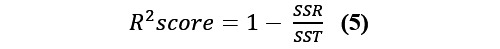





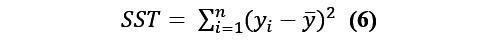





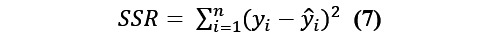



**Figure 4:**
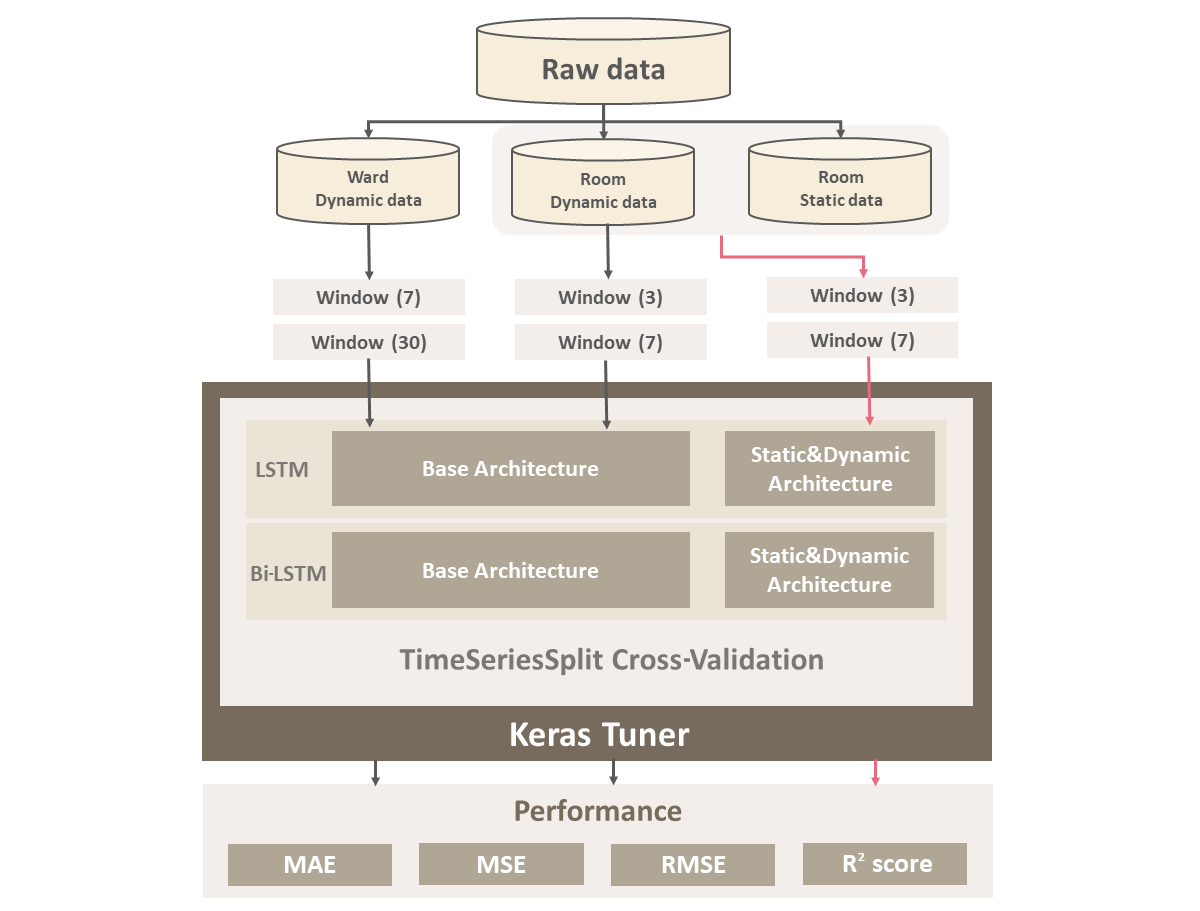
Overall flow in this study. Bi-LSTM: bidirectional long short-term memory; LSTM: long short-term memory; MAE: mean absolute error; MSE: mean square error; RMSE: root mean square error.

Here, SSR represents the sum of squared differences between the predicted and actual values, and SST represents the sum of squared differences between the actual values and the mean value of actual values. Figure 4 shows the prediction method and overall flow in this study.

## Results

We used 2 DL models, LSTM and Bi-LSTM, and compared the performance of 12 different prediction models. These models have been denoted as ward 7 days (W7D), ward 30 days (W30D), room 3 days (R3D), room 7 days (R7D), room static 3 days (RS3D), and room static 7 days (RS7D). Using Keras Tuner, we adjusted the hyperparameters of the models and subsequently validated the models through a 5-fold time series cross-validation.

The prediction performances of the models for WBOR and RBOR were compared, which showed that they were more accurate at predicting WBOR, with MAE values of 0.06 to 0.07. The W7D model based on Bi-LSTM, which used 7 days of ward data to predict the next day’s ward occupancy, had a MAE value of 0.067, MSE value of 0.009, and RMSE value of 0.094, showing high accuracy. The R^2^ score was also 0.544, which was approximately 0.240 higher than that of the W30D model (0.304), indicating that the variables in that model explained occupancy reasonably well.

We next compared the performances of the 8 models for RBOR prediction, and among them, the RS7D model based on Bi-LSTM, which was trained on a 7-day time step by integrating static and dynamic data, showed the best performance. It achieved a MAE value of 0.129, MSE value of 0.050, RMSE value of 0.227, and R^2^ score of 0.260. In particular, the R^2^ score outperformed that of the R3D model by 0.014. These data are summarized in Table 4. Regarding the WBOR prediction model, the model with a shorter training unit, W7D, demonstrated better performance. However, regarding the RBOR prediction model, the model with a longer training unit of 7 days, which incorporated detailed room-specific information, exhibited slightly higher performance than the model with a shorter training unit of 3 days. The model with the added room-specific information still demonstrated superior performance overall.

**Figure 5:**
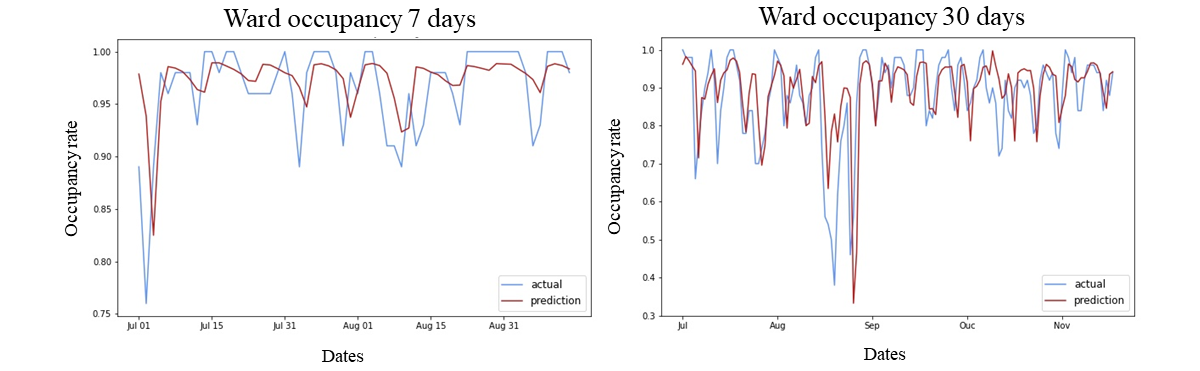
Examples of the predicted and actual bed occupancy rates for the 2-month period from July to August 2022 for ward 7 days and the 5-month period from July to November 2022 for ward 30 days.

We visualized the predicted and actual occupancy for Bi-LSTM models and investigated the occupancy trends since July 2022 on our test data set. First, we selected a specific ward in W7D to demonstrate the change in the WBOR over 2 months. The right panel of Figure 5 shows the WBOR change over 5 months from July 2022 in W30D. The blue line represents the actual occupancy value, and the red line represents the predicted occupancy value by the model. This provides an at-a-glance view of the overall predicted occupancy level for each month and allows hospital staff to observe trends to obtain a rough understanding of the WBOR.

**Figure 6:**
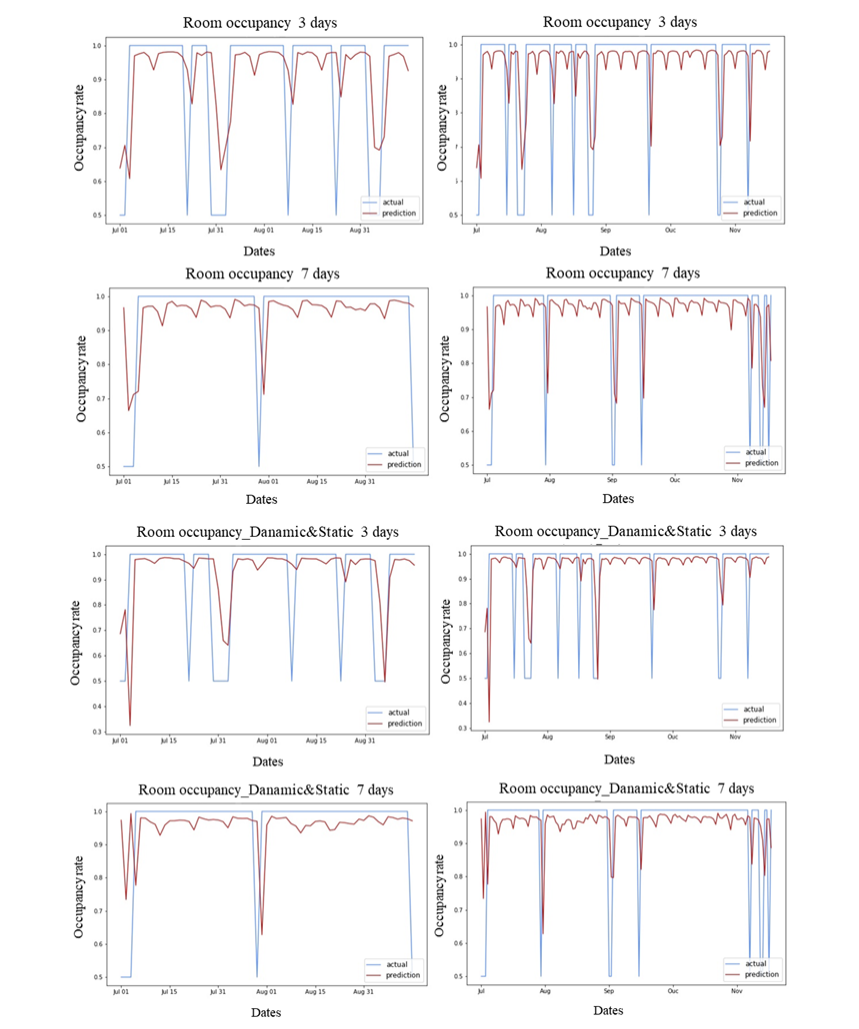
Examples of the predicted and actual bed occupancy rates for the 2-month period from July to August 2022 and the 5-month period from July to November 2022.

Figure 6 shows graphs of occupancy rate values for a randomized specific room, displaying the predicted and actual values for the 4 RBOR prediction models, with 2 graphs for each model. The left graph shows the occupancy rate change over 5 months from July to November 2022, and the right graph shows the occupancy rate for the months of July and August, providing a detailed view of the RBOR. By examining the trends of the predicted and actual values for the 4 models in this period for a specific room, we can observe that the models maintain a similar trend to the actual occupancy rate.

**Table 4:** Performances of the occupancy prediction models.

Model and fold			MAE^a^				MSE^b^			RMSE^c^				R^2^ score		
			LSTM^d^		Bi-LSTM^e^		LSTM	Bi-LSTM		LSTM		Bi-LSTM		LSTM		Bi-LSTM
**Ward**																
	**W30D^f^**															
		1	0.081	0.097		0.014		0.015	0.117		0.121		0.040		−0.081	
		2	0.074	0.064		0.011		0.007	0.107		0.085		0.106		0.430	
		3	0.118	0.109		0.031		0.025	0.175		0.161		−0.130		0.086	
		4	0.150	0.087		0.033		0.013	0.182		0.113		−0.572		0.399	
		5	0.087	0.061		0.019		0.008	0.139		0.089		0.212		0.678	
		Mean	0.102	0.084		0.021		0.014	0.144		0.114		−0.068		0.304	
	**W7D^g^**															
		1	0.071	0.063		0.011		0.007	0.103		0.086		0.263		0.479	
		2	0.067	0.054		0.009		0.005	0.094		0.071		0.302		0.606	
		3	0.119	0.091		0.033		0.016	0.183		0.126		−0.241		0.408	
		4	0.116	0.068		0.021		0.009	0.145		0.098		−0.009		0.537	
		5	0.083	0.060		0.015		0.007	0.123		0.087		0.380		0.690	
		Mean	0.091	0.067		0.018		0.009	0.130		0.094		0.139		0.544	
**Room**																
	**R7D^h^**															
		1	0.120	0.111		0.057		0.045	0.238		0.212		0.026		0.226	
		2	0.127	0.108		0.057		0.047	0.238		0.216		0.054		0.222	
		3	0.190	0.148		0.167		0.072	0.327		0.269		0.018		0.336	
		4	0.209	0.162		0.068		0.055	0.261		0.234		−0.089		0.125	
		5	0.158	0.124		0.069		0.048	0.263		0.220		0.102		0.370	
		Mean	0.161	0.131		0.071		0.053	0.265		0.230		0.022		0.256	
	**R3D^i^**															
		1	0.134	0.115		0.058		0.045	0.242		0.212		0.001		0.229	
		2	0.130	0.097		0.060		0.048	0.245		0.220		0.006		0.195	
		3	0.178	0.147		0.118		0.080	0.344		0.283		−0.084		0.266	
		4	0.210	0.204		0.078		0.075	0.280		0.275		−0.247		−0.201	
		5	0.161	0.120		0.064		0.048	0.254		0.220		0.168		0.377	
		Mean	0.163	0.167		0.076		0.059	0.273		0.242		−0.031		0.173	
	**RS7D^j^**															
		1	0.147	0.114		0.057		0.045	0.238		0.212		0.027		0.228	
		2	0.151	0.099		0.057		0.046	0.240		0.215		0.042		0.227	
		3	0.216	0.160		0.104		0.063	0.322		0.267		0.048		0.260	
		4	0.194	0.152		0.064		0.050	0.252		0.224		−0.016		0.198	
		5	0.181	0.120		0.068		0.047	0.261		0.217		0.112		0.385	
		Mean	0.178	0.129		0.070		0.050	0.262		0.227		0.043		0.260	
	**RS3D^k^**															
		1	0.109	0.116		0.056		0.046	0.237		0.215		0.039		0.213	
		2	0.118	0.092		0.061		0.048	0.246		0.219		−0.009		0.203	
		3	0.182	0.160		0.116		0.090	0.340		0.300		−0.062		0.172	
		4	0.278	0.191		0.152		0.065	0.389		0.255		−1.410		−0.039	
		5	0.159	0.116		0.074		0.047	0.272		0.218		0.043		0.387	
		Mean	0.169	0.135		0.092		0.059	0.297		0.241		−0.028		0.187	

^a^MAE: mean absolute error.

^b^MSE: mean square error.

^c^RMSE: root mean square error.

^d^LSTM: long short-term memory.

^e^Bi-LSTM: bidirectional long short-term memory.

^f^W30D: ward 30 days.

^g^W7D: ward 7 days.

^h^R7D: room 7 days.

^i^R3D: room 3 days.

^j^RS7D: room static 7 days.

^k^RS3D: room static 3 days.

## Discussion

### Principal Findings

The entire data set of this study consisted of administrative data collected at AMC at an hourly interval for each ward from May 27, 2020, to November 21, 2022. To improve the hospital’s challenges, we developed a model to predict the occupancy rate of wards and rooms. Our aim was to contribute toward administrative and financial planning for bed management within the hospital.

During the specified period, we compared the results of using DL models to predict the overall BOR for each ward and individual rooms. In the case of WBOR prediction, the MAE of the 7-day window model based on Bi-LSTM was approximately 0.067, demonstrating a remarkably close prediction to the occupancy compared with that of the 30-day window model based on LSTM, with a difference of approximately 0.035. Furthermore, the MSE and RMSE were 0.009 and 0.094, respectively, indicating high accuracy in the predictions. Moreover, the R^2^ score of 0.544 indicated that the model had better explanatory potential than the average. For the individual RBOR prediction, among the 8 models, the RS7D model based on Bi-LSTM performed the best, exhibiting a MAE of approximately 0.129, which was remarkably lower than that of the other models. Moreover, the MSE and RMSE were significantly lower than those of the RBOR models, with differences of 0.042 and 0.07, respectively. The R^2^ score of 0.260 indicated that it had higher explanatory potential than the RS3D models based on LSTM, with the value being higher by 0.291.

Finally, we visualized the predicted and actual values on a graph for a specific period and observed that each model captured the trend of the actual BOR quite well. Although the models were less accurate in predicting low occupancy periods, they followed the general trend closely. Overall, these findings demonstrate that our DL models effectively predicted BORs for both wards and individual rooms, with certain models demonstrating superior performance in different scenarios.

### Strengths and Limitations

Although the models in this study demonstrated good performance in following the trends of BORs and achieved good results, there were several limitations in this research. First, there were limitations in the data. Although we used administrative data and detailed room information available from the hospital to enable the models to capture occupancy trends, the relationship between the variables and the model’s explanatory potential showed room for improvement, as indicated by the R^2^ score. To achieve higher prediction accuracy, it would be beneficial to incorporate diverse data sources and real-time updated information.

Second, there was variability in external factors. Hospital BORs are heavily influenced by external environmental factors. Sudden events, such as environmental factors and outbreaks of infectious diseases like COVID-19, can render accurate prediction of bed occupancy challenging [18,32]. Furthermore, seasonal effects and accidents can increase the number of patients. Sufficient collection of long-term data on these external factors would be necessary, but such uncertainties can reduce the accuracy of predictions.

Despite these limitations, our study demonstrated a significant level of adherence to trends in the prediction of individual ward and room occupancy. More detailed variables and a longer period of data accumulation would be required to predict the specific number of beds.

### Conclusion

We presented models that can predict the occupancy rates of wards and individual hospital rooms using artificial neural networks based on time-series data. The predicted results of these models demonstrated a high level of accuracy in capturing the future trends of the BOR. In particular, we presented 8 RBOR models with structure and window changes to compare their performance and found that the RS7D model showed the best performance. Our results can be implemented as a web application on hospital online dashboards, as depicted in Figure 1 [25]. In fact, Johns Hopkins University has been applying these methods in their command center to monitor hospital capacity and achieve effectiveness in patient management planning [39].

Furthermore, predicting BORs supports patient admission and discharge planning, helping to alleviate overcrowding in emergency departments and reduce patient waiting times. Staff members can effectively schedule patient admission and discharge, and minimize waiting times by understanding the BOR, providing urgent treatment to emergency patients. Moreover, providing appropriate information to patients waiting in the emergency department can increase patient satisfaction and facilitate efficient transition to hospital admission [40,41]. By applying AI models that combine BOR prediction, which contributes toward reducing emergency department waiting times with individual patient admission and discharge prediction, hospitals can achieve resource optimization and cost savings, resulting in improved patient satisfaction.

## Abbreviations

AI: artificial intelligence
AMC: Asan Medical Center
Bi-LSTM: bidirectional long short-term memory
BOR: bed occupancy rate
DL: deep learning
DNN: deep neural network
LeakyReLU: leaky rectified linear unit
LSTM: long short-term memory
MAE: mean square error
ML: machine learning
R3D: room 3 days
R7D: room 7 days
RBOR: room bed occupancy rate
RMSE: root mean square error
RNN: recurrent neural network
RS3D: room static 3 days
RS7D: room static 7 days
W7D: ward 7 days
W30D: ward 30 days
WBOR: ward bed occupancy rate
